# PBK/TOPK Is a Favorable Prognostic Biomarker Correlated with Antitumor Immunity in Colon Cancers

**DOI:** 10.3390/biomedicines10020299

**Published:** 2022-01-27

**Authors:** Dong-Hee Lee, Yu-Jeong Jeong, Ju-Young Won, Hye-In Sim, Yoon Park, Hyung-Seung Jin

**Affiliations:** 1 Department of Convergence Medicine, Asan Institute for Life Sciences, Asan Medical Center, University of Ulsan College of Medicine, Seoul 05505, Korea; dhlee3342@gmail.com (D.-H.L.); yjrhdwn96@gmail.com (Y.-J.J.); wjo9956@gmail.com (J.-Y.W.); 2 Center for Theragnosis, Biomedical Research Institute, Korea Institute of Science and Technology (KIST), Seoul 02792, Korea; gpdls5541@naver.com

**Keywords:** PBK/TOPK, colon cancer, prognosis, immunotherapy, tumor mutation burden

## Abstract

Immune checkpoint inhibitor therapy has proven efficacy in a subset of colon cancer patients featuring a deficient DNA mismatch repair system or a high microsatellite instability profile. However, there is high demand for more effective biomarkers to expand the colon cancer population responding to ICI therapy. PBK/TOPK, a serine/threonine kinase, plays a role in cell cycle regulation and mitotic progression. Here, we investigated the correlation between PBK/TOPK expression and tumor immunity and its prognostic value in colon cancer. Based on large-scale bioinformatics analysis, we discovered that elevated PBK/TOPK expression predicted a favorable outcome in patients with colon cancer and was positively associated with immune infiltration levels of CD8+ T cells, CD4+ T cells, natural killer cells, and M1 macrophages. In contrast, a negative correlation was found between PBK/TOPK expression and immune suppressor cells, including regulatory T cells and M2 macrophages. Furthermore, the expression of PBK/TOPK was correlated with the expression of T-cell cytotoxicity genes in colon cancer. Additionally, high PBK/TOPK expression was associated with mutations in DNA damage repair genes, and thus with increased tumor mutation and neoantigen burden. These findings suggest that PBK/TOPK may serve as a prognostic and predictive biomarker for immunotherapy in colon cancer.

## 1. Introduction

Colon cancer is a malignant disease ranked third in cancer incidence and second in cancer mortality worldwide [1]. The incidence and mortality have been slowly declining each year, mainly owing to the surgical resection of primary tumors at the early localized stages. The 5-year survival rate at a late stage, however, remains profoundly low [1]. Targeted therapy, such as cetuximab or bevacizumab, has been shown to prolong overall survival (OS), but it is effective only in a subset of patients with colon cancer [2]. Two immune checkpoint inhibitors (ICIs) that target programmed cell death-1 (PD-1) have been FDA-approved for the treatment of patients with colon cancer exhibiting high microsatellite instability (MSI-H) or DNA mismatch repair (MMR) deficiency (dMMR) [3,4]. However, response to anti-PD-1 immunotherapy is highly variable, and tumor mutational burden (TMB) alone is insufficient to predict responses in colon cancers [5]. Therefore, the development of effective predictors of immunotherapeutic response for patients with colon cancer is crucial.

PDZ-binding kinase (PBK) (also known as T-lymphokine-activated killer cell-originated protein kinase (TOPK)) is a serine-threonine kinase aberrantly expressed in a variety of tumors [6]. PBK/TOPK activates downstream mitogen-activated protein kinases (MAPKs), such as an extracellular signaling-regulated kinase or p38 or Jun N-terminal kinase, resulting in tumorigenesis under different circumstances [7,8,9]. PBK/TOPK is involved in the regulation of cell cycle and apoptosis pathways [10,11,12,13]. Dysregulated PBK/TOPK expression promotes cancer growth and progression [14]. Considering its oncogenic roles, PBK/TOPK has been studied as a promising therapeutic target in cancer [6]. Previous studies have reported that high PBK/TOPK expression is correlated with a poor prognosis in colon, gastric, lung, and brain cancers [15,16,17,18,19]. However, in other studies, PBK/TOPK overexpression has shown a favorable prognosis in colon, bile duct, esophagus, and oral carcinomas [20,21,22,23,24,25]. Thus far, the discrepancy between the cellular mechanism and favorable prognosis has not been fully discussed; in particular, the prognostic significance of PBK/TOPK remains controversial in colon carcinomas.

It has become increasingly clear that the survival rate of patients with cancer is significantly associated with their immune profiles, such as immune cell composition and inflammatory signatures in the tumor microenvironment [26,27]. In this study, we investigated the prognostic role of PBK/TOPK in colon cancer, with a focus on genes involved in immunity. We found that high PBK/TOPK expression correlates with increased accumulation of antitumor immune cells and favorable prognosis in patients with colon cancer. Our findings indicate that PBK/TOPK may be a predictor of favorable prognosis and immunotherapeutic response for patients with colon cancer.

## 2. Materials and Methods

Gene expression and clinicopathological data of colon cancer patients and healthy volunteers were collected and obtained from public databases, as described below.

### 2.1. PBK/TOPK Expression Analysis

PBK/TOPK gene expression analyses of tumor and normal samples in The Cancer Genome Atlas (TCGA) and Genotype-Tissue Expression (GTEx) were performed on the GEPIA2 web portal (http://gepia2.cancer-pku.cn/, accessed on 13 October 2021) [28]. The gene expression data of 1372 Cancer Cell Line Encyclopedia (CCLE) cell lines were obtained from the Dependency Map (DepMap) Public 21Q3 dataset on the DepMap web portal (https://depmap.org/portal/, accessed on 15 September 2021). Normal tissue gene expression data were obtained from the GTEx portal (https://gtexportal.org/, accessed on 8 August 2021). PBK/TOPK mRNA expression of the CCLE and GTEx was visualized using the programming software R (version 4.1.1, https://www.r-project.org/, accessed on 8 August 2021). To confirm the expression profile of TCGA and GTEx, other gene expression data for colon tumor samples and paired normal samples were downloaded from the Gene Expression Omnibus (https://www.ncbi.nlm.nih.gov/geo/, accessed on 15 September 2021): GSE44076. In total, 98 pairs of tumor and normal tissue data were analyzed for differential PBK/TOPK expression. To analyze methylated CpG sites of the PBK/TOPK promoter, Human Methylation 450K data were downloaded from the UCSC Xena browser (https://xenabrowser.net/, accessed on 10 September 2020) [29]. Immunohistochemical images of PBK/TOPK protein expression were obtained from the Human Protein Atlas (http://www.proteinatlas.org, accessed on 14 October 2021).

### 2.2. Survival Analysis

Survival analysis between PBK/TOPK-high and -low groups was performed with the GEPIA2 web portal across TCGA cancer types [28]. Briefly, TCGA patient samples were sub-grouped into two cohorts according to the 25% cutoff expression values. The OS and disease-free survival (DFS) of the two cohorts were visualized using Kaplan–Meier curves with hazard ratios (HRs) of the Cox-PH model and log-rank *p* values. A meta-analysis was performed to assess the correlation between PBK/TOPK gene expression and prognosis across a variety of solid tumors. Prognosis data were downloaded from the PROGgeneV2 database (http://www.progtools.net/gene/, accessed on 27 July 2019) [30] and cohorts with sample numbers over 60 were included. HRs with 95% CI and log-rank *p* values across 54 cancer cohorts were displayed with a forest plot.

### 2.3. Immune Cell Infiltration Analysis

Immune cell infiltration scores were estimated with multiple deconvolution methods from TCGA and the Clinical Proteomic Tumor Analysis Consortium 2 (CPTAC-2) RNA sequencing data on the TIMER2.0 web portal (http://timer.cistrome.org/, accessed on 11 October 2021) [31]. TIMER [32], CIBERSORT [33], xCell [34], MCP-counter [35], quanTIseq [36], and EPIC [37] algorithms were used for the estimations. The analysis results were visualized using heatmaps or scatter plots with R (version 4.1.1, https://www.r-project.org/, accessed on 8 August 2021).

### 2.4. Differential Gene Expression and Correlation Analysis

RNA-sequencing gene expression and mutation data of TCGA and CPTAC-2 were downloaded from the GDC Data Portal (https://portal.gdc.cancer.gov/, accessed on 23 September 2021) using TCGAbiolinks R/Bioconductor package or the cBioPortal webpage [38,39]. For the differential expression (DE) gene analysis, TCGA or CPTAC-2 cohorts were divided into two subgroups according to the PBK/TOPK expression levels (“PBK/TOPK-high” and “PBK/TOPK-low” represented the cut-off values of the top and bottom 10% levels, respectively). A DE gene analysis between the two groups was performed using the limma R/Bioconductor package [40]. To investigate the pathway, a gene set enrichment analysis (GSEA) was conducted with MSigDB (version 7.4) using the fgsea R/Bioconductor package [41]. A pre-ranked gene matrix for GSEA was calculated using the limma *t*-statistic or Pearson’s correlation coefficient with PBK/TOPK.

### 2.5. Cell Culture and Generation of CRISPR-Cas9 Knockout (KO) Cell Lines

HCT-116 cells were purchased from the American Type Culture Collection. Cells were cultured in McCoy’s 5A medium (Thermo Fisher Scientific, Waltham, MA, USA) supplemented with 10% FBS (WELGENE, South Korea), 1% HEPES, and 100 units/mL penicillin-streptomycin. To generate PBK/TOPK-KO HCT-116 cells, CRISPR/Cas9-mediated DNA editing was performed. Both strands of oligo DNAs encoding sgRNAs that target *PBK* sequences were designed (5′-CAGAAGCTTGGCTTTGGTAC-3′, 5′-AGGCCGGGATATTTATAGT-3′). sgRNA oligonucleotides were annealed and cloned into PX461 and PX462 plasmids (Addgene, Cat #48140, #62987). HCT-116 cells were transfected with the plasmids and sgRNA-expressing cells were selected with puromycin. PBK/TOPK-KO clones were isolated by single-cell dilution cloning. KO clones were validated by Western blotting.

### 2.6. Immunoblotting

The antibody against PBK/TOPK was purchased from BD Biosciences (San Jose, CA, USA). Anti-GAPDH antibody was purchased from Santa Cruz Biotechnology (Dallas, TX, USA). Cells were lysed using an M-PER mammalian protein extraction reagent (Thermo Fisher Scientific, Waltham, MA, USA) supplemented with 1× Halt protease and phosphatase inhibitor cocktail (Thermo Fisher Scientific, Waltham, MA, USA). Proteins were separated on an SDS-polyacrylamide gel and transferred onto a PVDF membrane (MilliporeSigma, Burlington, MA, USA). The signals were developed using an enhanced chemiluminescence detection system (ElpisBio, South Korea) and visualized with ChemiDoc (Bio-Rad Laboratories, Hercules, CA, USA).

### 2.7. Cell Cycle Analysis

Wild-type (WT) and PBK/TOPK-KO HCT-116 cells were incubated for one day in a humidified incubator with 5% CO_2_ after seeding. To induce DNA damage, HCT-116 cells were treated with SN-38 (Selleckchem, Houston, TX, USA) or with vehicle at the indicated concentrations and time points. Cells were fixed in ice-cold 70% ethanol and stained with propidium iodide solution (BioLegend, San Diego, CA, USA). The cell cycle was analyzed using a CytoFLEX flow cytometer (Beckman Coulter, Brea, CA, USA).

### 2.8. Statistical Analysis

Statistical analysis was performed using the GraphPad Prism (version 9.2.0, La Jolla, CA, USA) software and R (version 4.1.1, https://www.r-project.org/, accessed on 8 August 2021). Significance was determined using the two-tailed paired *t*-test for comparing tumor and matched normal samples. Student’s *t*-test was used for the independent two-group comparison, one-way ANOVA was used for the multiple group comparison, and the log-rank test was used for the survival analysis. *p* values < 0.05 were considered statistically significant (* *p* < 0.05, ** *p* < 0.01, *** *p* < 0.001, and **** *p* < 0.0001).

## 3. Results

### 3.1. High PBK/TOPK Expression Shows a Good Prognosis in Colon Cancer

**Figure 1:**
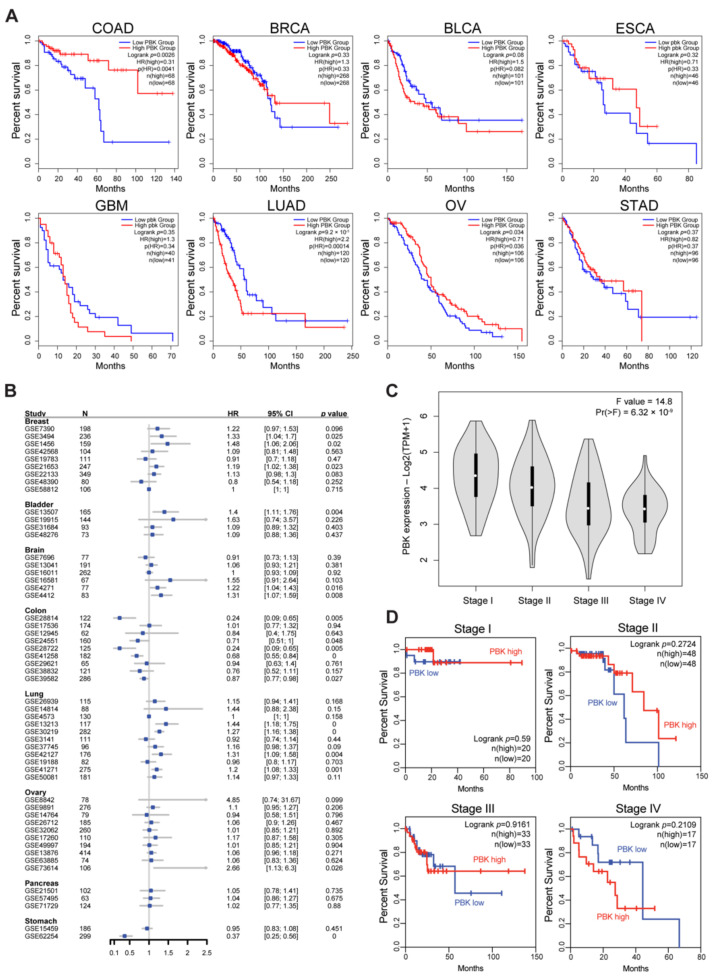
Positive prognostic value conferred by PBK/TOPK expression in colon cancer. Kaplan–Meier curves for overall survival (OS) analysis according to PBK/TOPK expression across a variety of solid tumors (**A**) and different stages of COAD (**D**) in TCGA. (**B**) Meta-analysis result of 54 solid tumor cohorts to assess OS according to PBK/TOPK expression. (**C**) PBK/TOPK gene expression level by tumor stages in TCGA COAD. Statistical significance is determined by one-way ANOVA. COAD, colon adenocarcinoma; BRCA, breast invasive carcinoma; BLCA, bladder urothelial carcinoma; ESCA, esophageal carcinoma; GBM, glioblastoma multiforme; LUAD, lung adenocarcinoma; OV, ovarian serous cystadenocarcinoma; STAD, stomach adenocarcinoma.

PBK/TOPK is known to be highly expressed on various malignant cells compared to normal tissues, which show a limited level of PBK/TOPK expression, except for the testes (Supplementary Figures S1 and S2) [6]. Analysis of TCGA data base revealed that the *PBK* promoter was hypo-methylated in colon cancer tissues compared with normal tissues, indicating that PBK/TOPK can be epigenetically induced in colon cancers (Supplementary Figure S3). We next examined the prognostic value of PBK/TOPK in colon adenocarcinoma (COAD), breast invasive carcinoma (BRCA), bladder urothelial carcinoma (BLCA), esophageal carcinoma (ESCA), glioblastoma multiforme, lung adenocarcinoma (LUAD), ovarian serous cystadenocarcinoma (OV), and stomach adenocarcinoma (STAD) of TCGA cohort where its tumorigenic roles are proposed [16,42,43,44,45,46,47,48]. High PBK/TOPK expression exhibited worse OS or DFS in BLCA (OS HR = 1.5, *p* = 0.08; DFS HR = 1.9, *p* = 0.015) and LUAD (OS HR = 2.2, *p* < 0.001; DFS HR = 2.4, *p* < 0.001). On the contrary, PBK/TOPK expression was correlated with an improved prognosis in COAD (OS HR = 0.31, *p* = 0.0026; DFS HR = 0.58, *p* = 0.12) and OV (OS HR = 0.71, *p* = 0.034; DFS HR = 0.82, *p* = 0.25) (Figure 1A and Supplementary Figure S4). Two upper gastrointestinal (GI) adenocarcinomas (ESCA and STAD) exhibited superior OS and DFS according to PBK/TOPK expression (ESCA OS HR = 0.71, DFS HR = 0.8; STAD OS HR = 0.82, DFS HR = 0.62); however, they did not reach statistical significance (Figure 1A and Supplementary Figure S4). Our meta-analysis of survival data further confirmed a positive correlation between PBK/TOPK expression and OS in colon cancers, but not in ovarian cancers (Figure 1B). We could not observe a correlation between PBK/TOPK expression and OS in ESCA and STAD owing to a lack of cohorts in the database. We next examined whether PBK/TOPK expression can differentiate patients with colon cancer exhibiting a different prognosis status. Since colon cancer progresses with a stepwise accumulation of genetic or epigenetic alterations and changes [49,50], we assessed PBK/TOPK expression levels in different stages of colon cancer (Figure 1C). PBK/TOPK expression gradually decreased as colon cancer progressed. The OS analysis results also demonstrated a correlation between a good prognosis and PBK/TOPK expression in stage I-III colon cancers (Figure 1D). Together with previous findings [20,21], our analysis supports that PBK/TOPK expression exhibits a favorable prognosis in patients with colon cancer.

### 3.2. PBK/TOPK Gene Expression Is Correlated with Increased Accumulation of Antitumor Immune Cells

**Figure 2:**
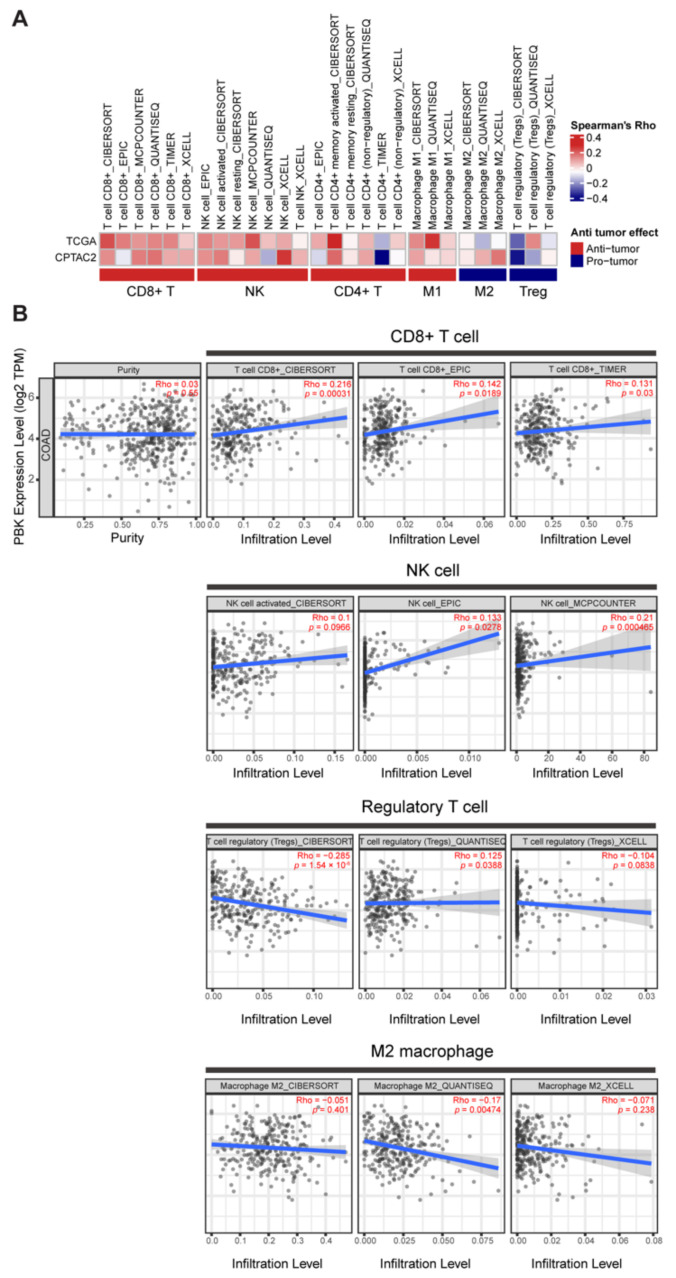
Correlation between PBK/TOPK expression and immune cell infiltration in colon cancer. (**A**) Correlation between the deconvolution analysis result and PBK/TOPK expression in TCGA and CPTAC-2 colon cancer samples. The heatmap plot represents Spearman’s correlation coefficients. (**B**) The scatter plot represents each correlation result illustrated in (**A**). Statistical significance was determined by Spearman’s correlation *p* value.

Immune cell infiltration is an important parameter for assessing tumor-immune interactions and the consequential effect on OS in colon cancer [51]. Hence, we examined whether a favorable prognosis of PBK/TOPK-high colon cancer was associated with increased accumulation of antitumor immune cells in the tumor microenvironment (TME). We analyzed the cellular composition of the intratumoral immune infiltrates with bulk RNA-sequencing data of TCGA and CPTAC-2 colon cancer by using different deconvolution algorithms in the TIMER2.0 web portal. The result showed a positive correlation between PBK/TOPK expression and the infiltration level of antitumor immune cells, including CD8+ T cells, natural killer (NK) cells, CD4+ T cells, and M1 macrophages. In addition, immunosuppressive protumor immune cells, such as regulatory T (Treg) cells and M2 macrophages, exhibited limited infiltration in PBK/TOPK-high patients with colon cancer (Figure 2A,B). The results were consistent over different deconvolution algorithms. We observed a similar correlation with PBK/TOPK expression when analyzing CD8+ T cell (R = 0.14, *p* = 0.0074), NK cell (R = 0.014, *p* = 0.79), and M1 macrophage scores (R = 0.12, *p* = 0.017) provided by Liu et al. (Supplementary Figure S5) [52].

### 3.3. Association between PBK/TOPK Expression and Antitumor Function of Tumor Infiltrating Immune Cells in Colon Cancers

**Figure 3:**
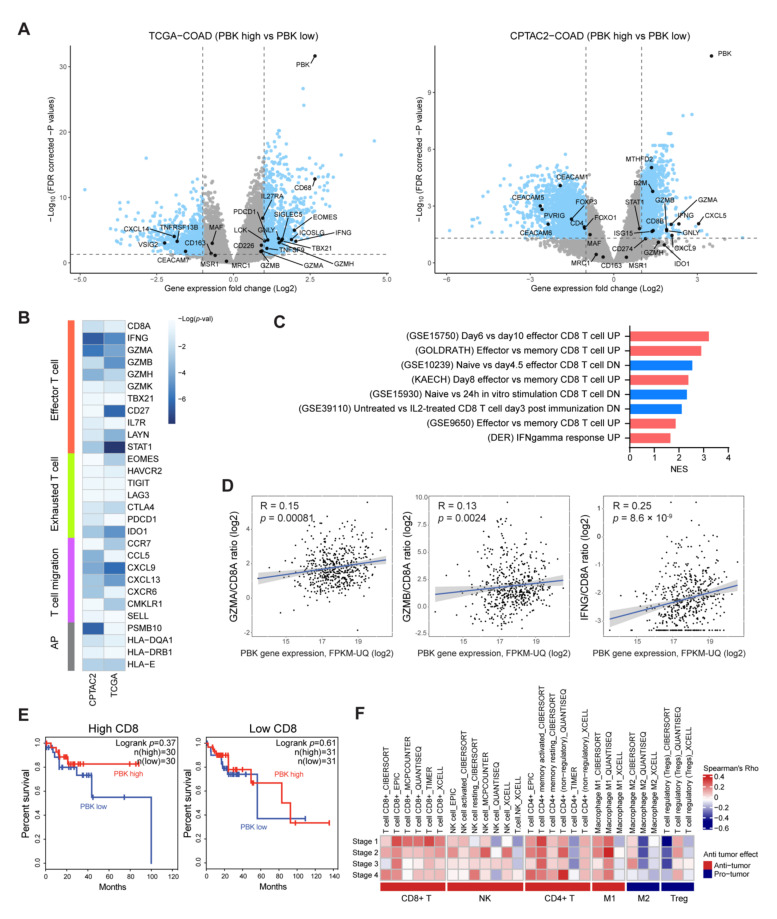
Expression of PBK/TOPK is positively correlated with T cell cytotoxic markers in colon cancers. (**A**) Volcano plots represent the DE gene analysis results between PBK/TOPK-high and -low groups in TCGA and CPTAC-2 colon cancer samples. Sky-blue dots represent DE genes by the cutoff of the absolute log2 fold change over one and the false discovery rate (FDR)-corrected *p* value under 0.05. (**B**) The heatmap plot represents the correlation between PBK/TOPK and each immune marker gene expression. AP, antigen presentation. *p* values were calculated from the *t*-statistic of Pearson’s correlation coefficients. (**C**) Significantly enriched pathways resulted from fgsea, performed with a pre-ranked gene matrix calculated with limma *t*-statistics in TCGA COAD DE gene analysis. Scarlet and blue bars represent up- and downregulated pathways, respectively. NES, normalized enrichment score. (**D**) Scatter plots represent the correlation between CD8A-normalized cytotoxic T-cell marker gene expression and PBK/TOPK expression. Statistical significance was determined based on Spearman’s correlation *p* value. (**E**) Kaplan–Meier curves of OS analysis according to PBK/TOPK expression in CD8-high and -low sub-cohorts in TCGA COAD. (**F**) The heatmap describes the immune cell infiltrating levels by tumor stages in TCGA patients with COAD.

To understand the functional characteristics of infiltrated immune cells in PBK/TOPK-high colon cancer, we performed a DE gene analysis between PBK/TOPK-high and -low groups. In total, 1802 (938 up- and 864 downregulated) and 3776 (1222 up- and 2554 downregulated) DE genes were analyzed in TCGA and CPTAC-2 colon cancer samples, respectively. First, we identified cytotoxic T cell markers (*IFNG*, *GNLY*, and *GZM* gene family), T-cell co-stimulatory molecules (*ICOSLG* and *TNFSF9*), T cell activation signaling genes (*LCK*, *TBX21*, *EOMES*, *MTHFD2*, and *ISG15*), T-cell migration genes (*CXCL* gene family), and a component of major histocompatibility complex (MHC) class I (*B2M*) in upregulated DE genes (Figure 3A). These findings suggest that high PBK/TOPK expression is positively correlated with cytotoxic and inflammatory immune signatures in colon cancer. On the contrary, T-cell inhibitory molecules (*PVRIG* and *CEACAM* gene family) and immunosuppressive Treg cell markers (*FOXP3*) were identified in downregulated DE genes (Figure 3A). We observed the downregulation of M2 macrophage markers (*CD163*, *MSR1*, *MAF*, and *MRC1*) but did not reach the DE gene criteria (absolute log2 fold change >1 and adjusted *p* value < 0.05). We confirmed that the DE gene analysis result was highly consistent with our immune cell infiltration analysis, as shown in Figure 2A. We next investigated the correlation between the expression of PBK/TOPK and genes related to the effector T cell, exhausted T cell, T-cell migration, and antigen presentation. Figure 3B shows that PBK/TOPK expression is significantly associated with the genes expressed in cytotoxic T cells, but not in the exhausted T cells. Furthermore, a positive correlation between the expression of effector T-cell-attracting chemokines (*CCL5* and *CXCL9*) and PBK/TOPK was observed. We also identified that the analyzed DE genes shown in Figure 3A were enriched in pathways related to “effector CD8 T cell up”, “naïve CD8 T cell down”, and “IFNgamma response up” with statistical significance (*p* value < 0.05) (Figure 3C). PBK/TOPK expression had significant positive correlations with the *GZMA/CD8A* (R = 0.15, *p* = 0.00081), *GZMB/CD8A* (R = 0.13, *p* = 0.0024), and *IFNG/CD8A* (R = 0.25, *p* = 8.6 × 10^−9^) ratio, suggesting that higher expression of PBK/TOPK is positively correlated with increased cytotoxic activity of CD8+ T cells (Figure 3D). Since PBK/TOPK expression was correlated with not only the degree of CD8+ cell infiltration but also their cytotoxic functions, we considered whether PBK/TOPK expression could represent an antitumor response by CD8+ T cells. We re-analyzed the prognostic value of PBK/TOPK in TCGA COAD cohort. First, we divided the entire cohort into two sub-cohorts: CD8-high and -low, according to *CD8A* and *CD8B* gene expression. We then examined the prognostic value of PBK/TOPK in each sub-cohort. In the CD8-low sub-cohort, patients with high PBK/TOPK expression did not show an improved prognosis compared with those with low PBK/TOPK expression. In the CD8-high sub-cohort, on the contrary, high PBK/TOPK expression was associated with a survival benefit in patients with colon cancer (Figure 3E), although the result did not reach statistical significance owing to the small number of patient samples. We next investigated the correlation between PBK/TOPK expression and immune cell infiltration level by tumor stage. The analysis illustrated that a good prognosis of PBK/TOPK was consistent with the degree of immune cell infiltration in stage I–III patients (Figure 1D and Figure 3F). Additionally, the correlation between PBK/TOPK expression and CD8+ T cell infiltration tended to decrease with increasing disease stage, suggesting that CD8+ T cells might be the major immune subtype determining the prognostic value of PBK/TOPK (Figure 3F) [53]. However, a favorable prognosis of PBK/TOPK in stage IV patients was not observed (Figure 1D and Figure 3F), likely because there exists a more important prognosis-determining factor than immune cell infiltration.

### 3.4. Positive Correlation between High PBK/TOPK Expression and Tumor Mutation Burden

**Figure 4:**
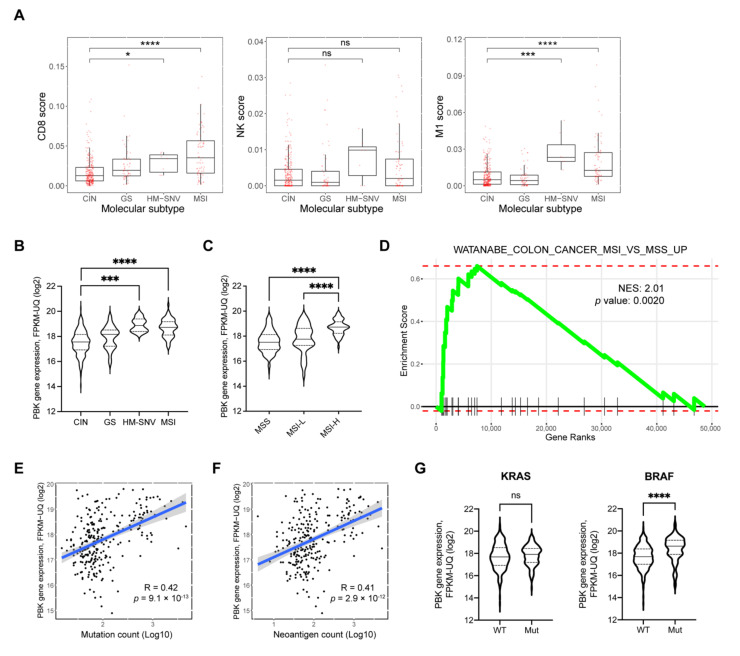
PBK/TOPK is associated with MSI and TMB in colon cancers. (**A**) Tumor molecular subtype analysis with CD8+ T cell, NK cell, and M2 macrophage scores in TCGA COAD samples. Molecular subtypes: CIN, chromosomal instability; GS, genome stable; HM-SNV, hypermutated-single-nucleotide variant predominant; MSI, microsatellite instability. (**B**) Violin plots showing the PBK/TOPK gene expression by molecular subtypes and (**C**) MSI status determined by the Bethesda panel in TCGA COAD. Statistical significance was determined by a two-tailed Student’s *t*-test. (**D**) Enrichment plot from fgsea performed with a gene matrix pre-ranked by Pearson’s *t*-statistics with PBK/TOPK co-expression. A positive correlation of PBK/TOPK expression with (**E**) total mutation counts and (**F**) neoantigen counts in TCGA COAD. The neoantigen count was conducted on The Cancer Immunome Atlas (TCIA) website (https://tcia.at/neoantigens, accessed on 16 August 2019). Statistical significance was determined by Spearman’s correlation *p* value. (**G**) Violin plots for comparing PBK/TOPK expression between wild-type (WT) and mutated (Mut) genes in TCGA COAD samples. Each *KRAS* and *BRAF* mutation represents G12/G13 and V600E, respectively. Statistical significance was determined by a two-tailed Student’s *t*-test. * *p* < 0.05, *** *p* < 0.001, **** *p* < 0.0001, ns, not significant.

Recently, Liu et al. suggested five molecular subtypes of adenocarcinomas of the GI tract: chromosomal instability (CIN), Epstein–Barr virus-positive, genome stable (GS), hypermutated-single-nucleotide variant (SNV) predominant (HM-SNV), and MSI [54]. Since both MSI and HM-SNV tumors are characterized by high T-cell infiltration and mutation burden in COAD, we sub-analyzed the degrees of CD8+ T cell, NK cell, and M1 macrophage infiltration in COAD. HM-SNV and MSI subtypes exhibited overall increases in the CD8+ T cell, NK cell, and M1 macrophage scores compared with the CIN subtype (Figure 4A). We next analyzed the PBK/TOPK expression level across four different tumor types of COAD. PBK/TOPK expression was significantly higher in HM-SNV and MSI types compared with the CIN type (Figure 4B). We confirmed the result with another MSI classification guideline by the Bethesda panel, indicating that MSI-H is associated with high PBK/TOPK expression compared with microsatellite stable (MSS) and MSI-low in TCGA COAD (Figure 4C). We obtained a similar result in the CPTAC-2 cohort (Supplementary Figure S6A). We conducted GSEA to confirm the correlation between PBK/TOPK expression and MSI status. As shown in Figure 4D, PBK/TOPK co-expressed genes were enriched in the gene set that represents a colon cancer MSI signature. Both HM-SNV and MSI showed hypermutated phenotypes due to defects in DNA repair pathways. We investigated whether PBK/TOPK is related to TMB in TCGA and CPTAC-2 COAD. PBK/TOPK expression was strongly correlated with the total mutation count (TCGA R = 0.42, *p* = 9.1 × 10^−13^; CPTAC-2 R = 0.45, *p* = 1.6 × 10^−6^) (Figure 4E and Supplementary Figure S6B). Similar to the total mutation count, the predicted neoantigen count also exhibited a strong positive correlation (R = 0.41, *p* = 2.9 × 10^−12^) with PBK/TOPK expression (Figure 4F). We next examined the association of the PBK/TOPK expression level with *KRAS* or *BRAF* mutations. These mutations are known to be crucial in colon cancer pathogenesis. MSI colon cancers with epigenetic silencing of the *MLH1* gene are correlated with the BRAF (V600E) mutation, while the KRAS hotspot mutation is greatly associated with MSS tumors and a subset of MSI tumors without defects in *MLH1* or *MSH2* genes [54]. PBK/TOPK showed a significant correlation only with the BRAF (V600E) but not KRAS (G12/G13) mutation (Figure 4G). These results reinforce our findings demonstrating the correlation between PBK/TOPK and MSI colon cancers.

### 3.5. PBK/TOPK Is Associated with DNA Repair Pathways

**Figure 5:**
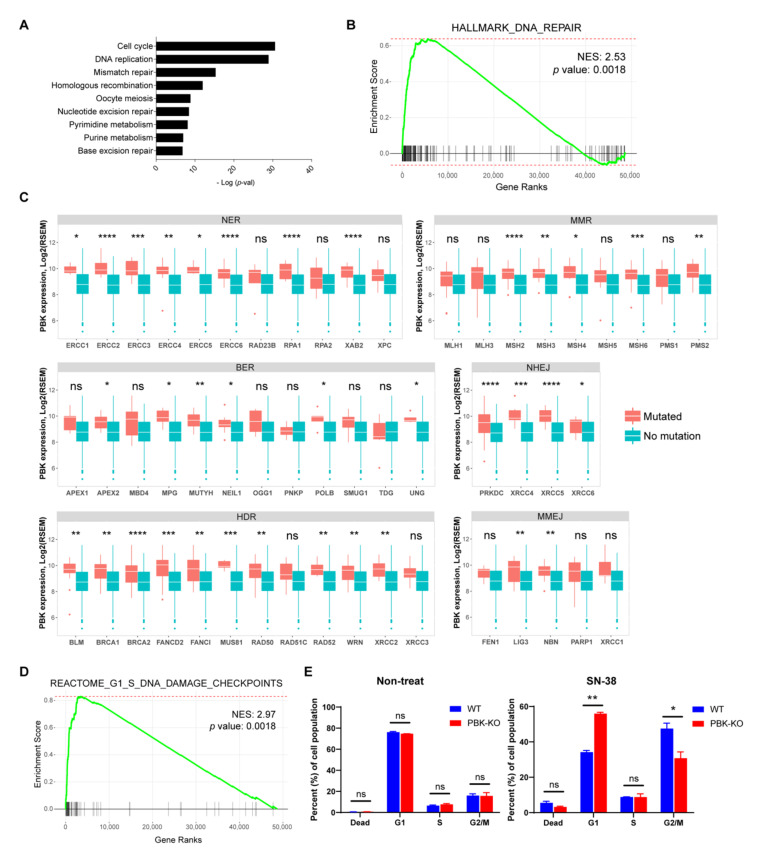
PBK/TOPK is associated with various DNA repair pathways. (**A**) Meta-analysis of gene expression array databases. The bar plot illustrates statistically significant Kyoto Encyclopedia of Genes and Genomes (KEGG) pathways concordant with PBK/TOPK. (**B**,**D**) Enrichment plot from fgsea performed with a gene matrix pre-ranked by Pearson’s *t*-statistics with PBK/TOPK expression. (**C**) Boxplots comparing PBK expression between WT and mutations of the indicated genes in TCGA COAD samples. Statistical significance was determined using a two-tailed Student’s *t*-test. (**E**) WT and PBK/TOPK-KO HCT-116 cells were treated with 1 nM of SN-38 for 48 h. Treated cells were stained with propidium iodide and analyzed by a flow cytometer. The data are representative of two independent experiments, and values are expressed as mean ± standard deviation. NER, nuclear excision repair; BER, base excision repair; HDR, homology-directed repair; MMR, mismatch repair; NHEJ, non-homologous end joining; MMEJ, microhomology-mediated end-joining. * *p* < 0.05, ** *p* < 0.01, *** *p* < 0.001, **** *p* < 0.0001, ns, not significant.

We next conducted a meta-analysis of gene expression array databases with the CO-Regulation Database tool (http://cord-db.org/, accessed on 31 July 2019) [55]. As shown in Figure 5A, PBK/TOPK was significantly concordant with several mitosis-related pathways, such as “cell cycle”, “DNA replication”, “pyrimidine metabolism”, and “purine metabolism”, in-line with the findings of previous studies [12,43]. Pathways related to DNA repair were also identified, including “mismatch repair”, “homologous recombination”, “nucleotide excision repair”, and “base excision repair”. We confirmed the results with GSEA. Genes that were co-expressed with PBK/TOPK were enriched in several DNA repair pathways, including the “hallmark DNA repair” gene set of MSigDB (Figure 5B). The analysis suggested that PBK/TOPK may play a role in the DNA repair mechanism.

MSI is caused by the defects of MMR genes, resulting in a high rate of SNVs and indel mutations. HM-SNV was characterized as a hyper-single nucleotide mutation with impairment of *POLE* and *POLD1* genes [54,56]. In addition to MMR genes, *POLE* and *POLD1* are related to multiple DNA repair pathways, such as nuclear excision repair (NER), base excision repair (BER), and double-strand break repair pathway as well as MMR [57].

Thus, we analyzed the correlations between PBK/TOPK expression and mutations of key genes that function in six different DNA repair pathways: NER, BER, homology-directed repair (HDR), non-homologous end joining (NHEJ), microhomology-mediated end-joining (MMEJ), and MMR in TCGA and CPTAC-2 COAD. PBK/TOPK-high colon cancers are more likely to have mutations in several DNA damage repair genes. PBK/TOPK expression showed strong correlations with mutation of genes involved in NER (*ERCC1*, *ERCC2*, *ERCC3*, *ERCC4*, *ERCC5*, *ERCC6*, *RPA1* and *XAB2*), BER (*APEX2, MPG*, *MUTYH*, *NEIL1*, *POLB* and *UNG)*, HDR (*BLM*, *BRCA1*, *BRCA2*, *FANCD2*, *FANCI*, *MUS81*, *RAD50*, *RAD51C*, *XRCC2* and *XRCC3*), NHEJ (*XRCC4*, *XRCC5*, *XRCC6*, and *PRKDC*), and MMEJ (*LIG3* and *NBN*) pathways (Figure 5C). PBK/TOPK expression was also significantly correlated with mutations of MMR genes (*MSH2*, *MSH3*, *MSH4*, *MSH6* and *PMS2*), supporting the association between PBK/TOPK and MSI colon cancer.

In addition, we found that the “Reactome G1/S DNA damage checkpoint” gene set was co-expressed with PBK/TOPK (Figure 5D). Since PBK/TOPK expression shows high correlations with TMB and DNA repair pathways, we investigated whether the upregulation of PBK/TOPK could enable the cells with DNA damage to bypass cell cycle checkpoints, leading to the accumulation of DNA mutations. We generated PBK/TOPK-KO HCT-116 colon cancer cell lines (Supplementary Figure S7). Knockouts of PBK/TOPK did not affect the rate of cell proliferation under normal culture conditions. However, a significant arrest in the G1 phase was observed in PBK/TOPK-KO HCT-116 cells upon treatment with the DNA-damaging agent SN-38 (an active metabolite of irinotecan) (Figure 5E), suggesting a possible role of PBK/TOPK at the G1/S checkpoint in response to DNA damage.

## 4. Discussion

Although anti-PD-1/PD-L1 antibodies may be effective in treating cancer, their response rates are only approximately 10% to 20% in unselected patients [58]. These response rates could be increased with patient selection. In metastatic CRC patients treated with pembrolizumab (anti-PD-1 antibody), patients with MMR-deficient tumors had a 40% response rate, compared to 0% for those with MMR-proficient tumors. However, only approximately 2–4% of patients with metastatic CRC have dMMR/MSI-high disease and some of them do not respond to anti-PD-1 therapy [59]. Therefore, it is important to discover more effective predictive biomarkers to improve treatment outcomes and extend the benefit of immunotherapy to a greater population of patients with CRC.

**Figure 6:**
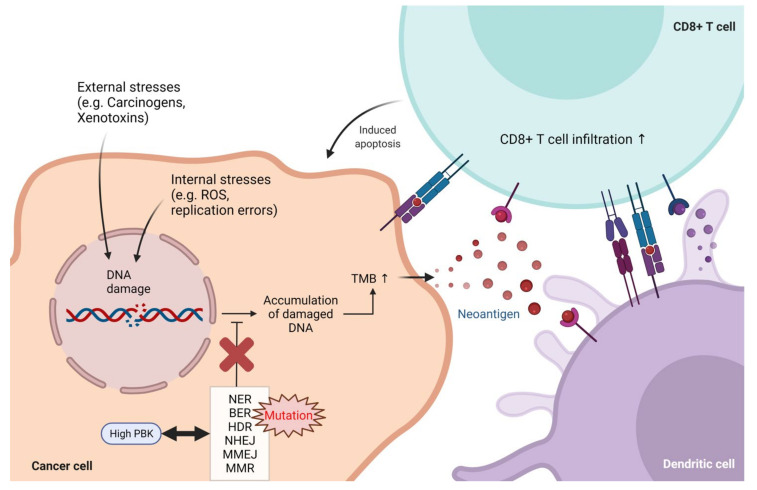
Proposed mechanism for the role of PBK/TOPK to induce tumor immunity in colon cancer. Damaged DNA is repaired by various repair mechanisms: NER, BER, HDR, NHEJ, MMEJ, and MMR. High PBK/TOPK expression has a positive correlation with mutations in DNA repair genes. These mutations induce the accumulation of damaged DNA, thereby increasing TMB and tumor-specific neoantigens, which are associated with increased tumor infiltration by cytotoxic immune cells. ROS, reactive oxygen species; TMB, tumor mutational burden; NER, nuclear excision repair; BER, base excision repair; MMR, mismatch repair; HDR, homology-directed repair; NHEJ, non-homologous end joining; MMEJ, microhomology-mediated end-joining.

In this study, we conducted a large-scale bioinformatic analysis of the genetic and clinicopathologic data of patients with colon cancer and revealed that PBK/TOPK could act as a favorable prognostic biomarker in colon cancer. Given the oncogenic role of PBK/TOPK in the process of tumorigenesis and tumor progression, it is interesting to find that high expression of PBK/TOPK correlates with an improved prognosis in colon cancer. Since tumor infiltrating immune cells contributes to determining the prognosis of colon cancer [60,61], we analyzed the changes in immune profiles including immune cell composition and inflammatory signatures. High PBK/TOPK expression in colon cancer was correlated with an increased infiltration level of cytotoxic immune cells, whereas activated CD8+ T- and NK-cell-infiltrated tumors also exhibited decreased infiltration of Treg cells and M2 macrophages that have immunosuppressive roles in TME. High cytotoxic activity, CD8/Treg ratios, and expression of IFN-γ and granzymes by CD8+ T cells were observed in PBK/TOPK-high colon cancer. Using genomic data drawn from TCGA and CPTAC-2, we found that high PBK/TOPK expression is associated with impaired DNA repair. Mutations in DNA repair genes were enriched in PBK/TOPK-high colon cancers. It has long been known that mutations in DNA repair pathways are associated with higher TMB and neoantigen load [62]. We found that colon cancer patients with high PBK/TOPK expression showed higher MSI and TMB, which is associated with neoantigen burden. Therefore, PBK/TOPK expression levels could serve as a surrogate biomarker for identifying colon cancer patients with high TMB. Further studies are required to determine if analyzing the expression level of PBK/TOPK in colon cancer may be a clinically viable method for predicting TMB levels. Owing to their increased tumor mutational burden, PBK/TOPK-high colon cancers could present neoantigens on MHC-I molecules and promote high levels of neoantigen specific T-cell activation, ultimately leading to the destruction of cancer cells (Figure 6).

Although PBK/TOPK is also overexpressed in most tumors, we did not observe a favorable prognosis in patients with other types of tumors except COAD. Previous studies have reported that high PBK expression is associated with a poor prognosis in patients with several types of malignancies, such as kidney renal clear cell carcinoma (KIRC), lower grade glioma (LGG), and liver hepatocellular carcinoma (LIHC) [63]. To address this discrepancy, we analyzed the correlation between PBK/TOPK expression and the degree of immune cell infiltration in non-small cell lung cancer (NSCLC). In two types of NSCLC, LUAD and lung squamous cell carcinoma (LUSC), the analysis exhibited a positive correlation of PBK/TOPK expression and a certain degree of immune cell infiltration (Supplementary Figure S8A,B). These observations are similar to those of colon cancer. However, a high PBK/TOPK expression was correlated with a poor prognosis in LUAD (Figure 1A and Supplementary Figure S4) and a good prognosis in LUSC (Supplementary Figure S8C,D). Another report demonstrated that PBK/TOPK-high esophageal squamous cell carcinoma showed a good prognosis; however, no immune cell infiltration was observed [23]. To understand these differences between cancer types, a comprehensive analysis is needed across all types of cancers with multiple prognostic factors, such as oncogene and tumor suppressor gene mutations, lineage-dependent gene expression, and specific immune cell subtypes. PBK/TOPK appears to promote mitosis for cancer cell proliferation according to our pathway analysis data (data not shown) and those of previous reports [6]; however, how much or how independently PBK/TOPK contributes to the proliferation of colon cancer cells is not clear. A CRISPR-based KO study showed that the expression of PBK/TOPK is not critical for cancer cell proliferation [64]. The dependency scores in the DepMap database also demonstrated that PBK/TOPK may not affect the proliferation of colon cancer cell lines (data not shown). In addition, PBK/TOPK-KO mice do not exhibit any differences in body weight or male reproductive systems [65]. Although these findings provide some support as to why PBK/TOPK upregulation is not associated with a significantly poor prognosis in most cancer types, further studies should be performed to unveil the exact roles of PBK/TOPK under different circumstances. Given that this study has a limited patient cohort size, additional data from prospective studies are needed.

## 5. Conclusions

A variety of biomarker strategies for ICI therapy have been extensively explored to identify patient populations that will respond to treatment [66]. In this study, we proposed the association between high PBK/TOPK expression and favorable prognose in patients with colon cancer. Given the correlation among MSI-H, the mutational load, and cytotoxic immune cell infiltration, upregulation of PBK/TOPK in colon cancer could be a potential candidate marker to guide patient selection for immunotherapy.

## Data Availability

All the data are available online with common access.

## Supplementary Materials

The following are available online at https://www.mdpi.com/article/10.3390/biomedicines10020299/s1, Figure S1: PBK/TOPK gene expression in various primary tumors, cell lines, and normal tissues, Figure S2: PBK/TOPK protein expression in colon cancer patient tissue samples, Figure S3: The methylation status of *PBK* promoter region in TCGA COAD and matched normal samples, Figure S4: Prognostic values of PBK/TOPK in various cancer types, Figure S5: Correlation between PBK/TOPK expression and immune cell infiltration in colon cancer, Figure S6: Correlation between PBK/TOPK expression and MSI subtype in the CPTAC-2 colon cancer samples, Figure S7: PBK/TOPK gene knockout in the HCT-116 colon cancer cell line, Figure S8: Correlation of PBK/TOPK expression with immune cell infiltration level and prognosis in lung cancer.
