# Supplementary material for: PBK/TOPK Is a Favorable Prognostic Biomarker Correlated with Antitumor Immunity in Colon Cancers
Source: Biomedicines. 2022 Jan 27;10(2):299. doi: 10.3390/biomedicines10020299 (PMC8869639; doi:10.3390/biomedicines10020299)
Supplement: Supplementary file 1 [file biomedicines-10-00299-s001.zip › biomedicines-1533518-supplementary.pdf]

Supplementary materials:

Supplementary Figure S1 to Supplementary Figure S8

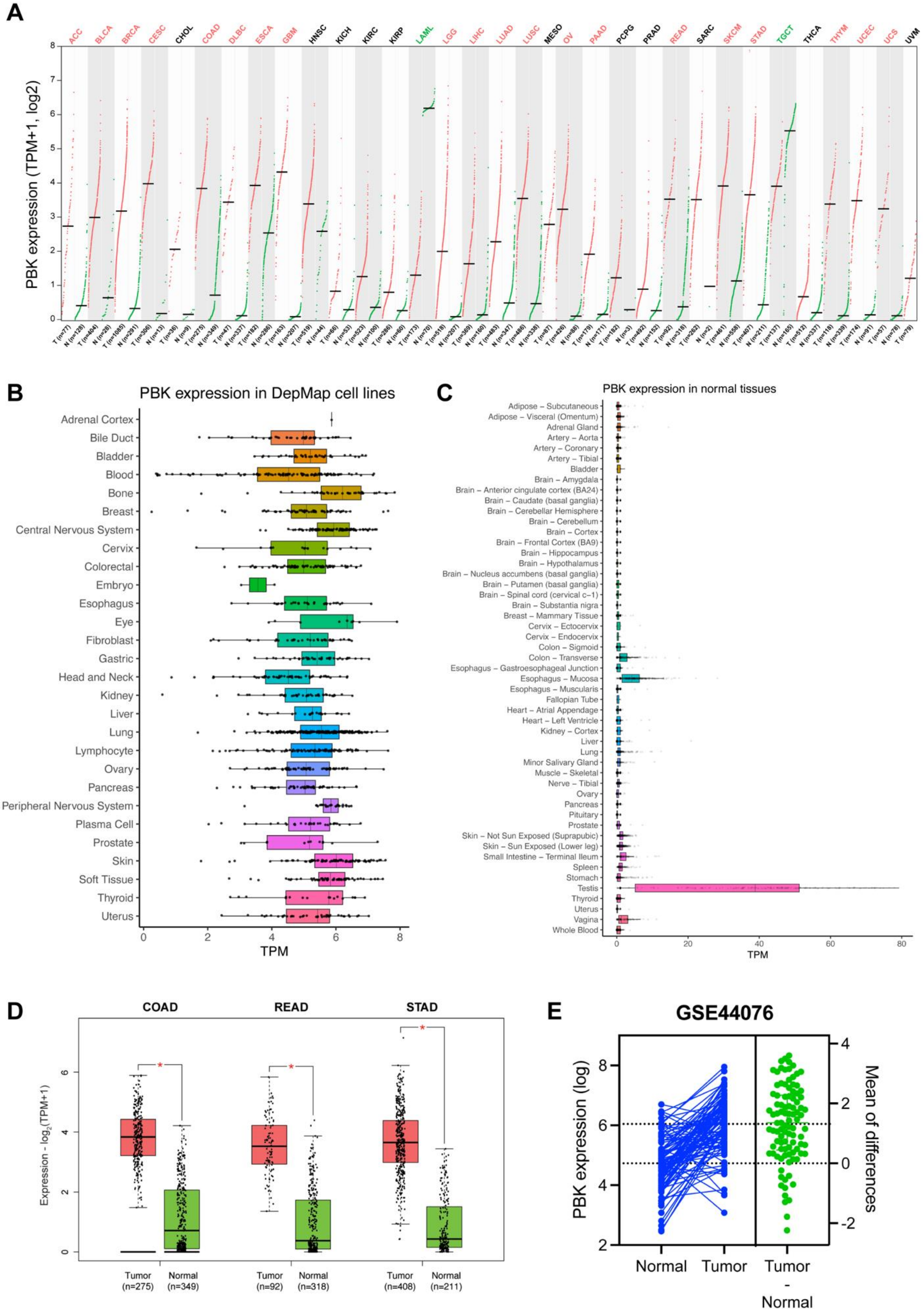
A
ACC
BLCA
BRCA
CESC
CHOL
COAD
DLBC
ESCA
GBM
HNSC
KICH
KIRC
KIRP
LAML
LGG
LIHC
LUAD
LUSC
MESO
OV
PAAD
PCPG
PRAD
READ
SARC
SKCM
STAD
TGCT
THCA
THYM
UCEC
UCS
UVM
PBK expression (TPM+1, log2)
B
PBK expression in DepMap cell lines
Adrenal Cortex
Bile Duct
Bladder
Blood
Bone
Breast
Central Nervous System
Cervix
Colorectal
Embryo
Esophagus
Eye
Fibroblast
Gastric
Head and Neck
Kidney
Liver
Lung
Lymphocyte
Ovary
Pancreas
Peripheral Nervous System
Plasma Cell
Prostate
Skin
Soft Tissue
Thyroid
Uterus
TPM
C
PBK expression in normal tissues
Adipose – Subcutaneous
Adipose – Visceral (Omentum)
Adrenal Gland
Artery – Aorta
Artery – Coronary
Artery – Tibial
Bladder
Brain – Amygdala
Brain – Anterior cingulate cortex (BA24)
Brain – Caudate (basal ganglia)
Brain – Cerebellar Hemisphere
Brain – Cerebellum
Brain – Cortex
Brain – Frontal Cortex (BA9)
Brain – Hippocampus
Brain – Hypothalamus
Brain – Nucleus accumbens (basal ganglia)
Brain – Putamen (basal ganglia)
Brain – Spinal cord (cervical c-1)
Brain – Substantia nigra
Breast – Mammary Tissue
Cervix – Ectocervix
Cervix – Endocervix
Colon – Sigmoid
Colon – Transverse
Esophagus – Gastroesophageal Junction
Esophagus – Mucosa
Esophagus – Muscularis
Fallopian Tube
Heart – Atrial Appendage
Heart – Left Ventricle
Kidney – Cortex
Liver
Lung
Minor Salivary Gland
Muscle – Skeletal
Nerve – Tibial
Ovary
Pancreas
Pituitary
Prostate
Skin – Not Sun Exposed (Suprapubic)
Skin – Sun Exposed (Lower leg)
Small Intestine – Terminal Ileum
Spleen
Stomach
Testis
Thyroid
Uterus
Vagina
Whole Blood
TPM
D
COAD
READ
STAD
Expression - log2(TPM+1)
Tumor (n=275)
Normal (n=349)
Tumor (n=92)
Normal (n=318)
Tumor (n=408)
Normal (n=211)
E
GSE44076
PBK expression (log)
Mean of differences
Normal
Tumor
Tumor - Normal

Supplementary Figure S1. PBK/TOPK gene expression in various primary tumors, cell lines, and normal tissues. **(A)** Pan-cancer expression analysis of the PBK/TOPK gene was performed with the GEPIA2 website. Tumor tissues (T, red dots) represent TCGA tumors. Normal tissues (N, green dots) represent TCGA and GTEx normal tissues. Expression values are presented as log-normalized transcripts per million (TPM) with median values (horizontal black bar). The red and green colors of the cancer type abbreviations denote that PBK/TOPK gene expression is significantly higher or lower in these tumor tissues compared to normal tissues, respectively. **(B)** Pan-cancer cell line expression data of the PBK/TOPK gene were obtained from the DepMap Public 21Q3 dataset on the DepMap website. The PBK/TOPK expression level was visualized by primary sites of cancer cell lines using R software. **(C)** PBK/TOPK gene expression data in each normal tissue were downloaded from the GTEx web portal. The PBK/TOPK expression level was visualized using R software. **(D)** Boxplots represent the PBK/TOPK gene expression in three gastrointestinal adenocarcinomas and corresponding normal tissues. Expression analysis was performed with the GEPIA2 website. **(E)** PBK/TOPK gene expression analysis between colon cancer and paired normal tissues. Expression data were downloaded from the GEO database. TCGA abbreviations: ACC, adrenocortical carcinoma; BLCA, bladder urothelial carcinoma; BRCA, breast invasive carcinoma; CESC, cervical squamous cell carcinoma and endocervical adenocarcinoma; CHOL, cholangiocarcinoma; COAD, colon adenocarcinoma; DLBC, diffuse large B-cell lymphoma; ESCA, esophageal carcinoma; GBM, glioblastoma multiforme; HNSC, head and neck squamous cell carcinoma; KICH, kidney chromophobe; KIRC, kidney renal clear cell carcinoma; KIRP, kidney renal papillary cell carcinoma; LAML, acute myeloid leukemia; LGG, lower grade glioma; LIHC, liver hepatocellular carcinoma; LUAD, lung adenocarcinoma; LUSC, lung squamous cell carcinoma; MESO, mesothelioma; OV, ovarian serous cystadenocarcinoma; PAAD, pancreatic adenocarcinoma; PCPG, pheochromocytoma and paraganglioma; PRAD, prostate adenocarcinoma; READ, rectum adenocarcinoma; SARC, sarcoma; SKCM, skin cutaneous melanoma; STAD, stomach adenocarcinoma; TGCT, testicular germ cell tumors; THCA, thyroid carcinoma; THYM, thymoma; UCEC, uterine corpus endometrial carcinoma; UCS, uterine carcinosarcoma; UVM, uveal melanoma.

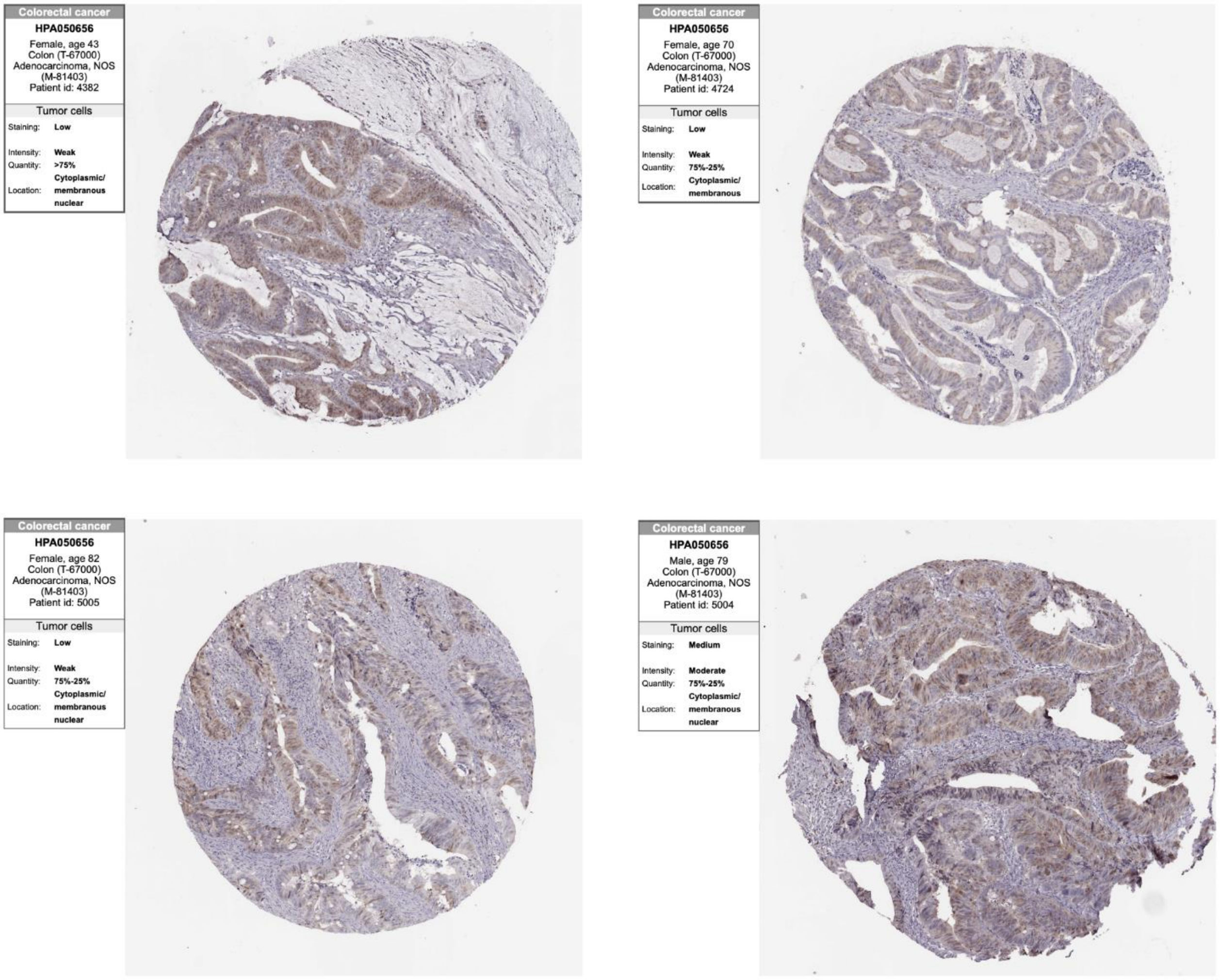

Supplementary Figure S2. PBK/TOPK protein expression in colon cancer patient tissue samples. Colon cancer tissue images were downloaded from the Human Protein Atlas website. PBK/TOPK was stained with the HPA050656 antibody. Low and medium staining images of four patients with colon adenocarcinoma are shown.

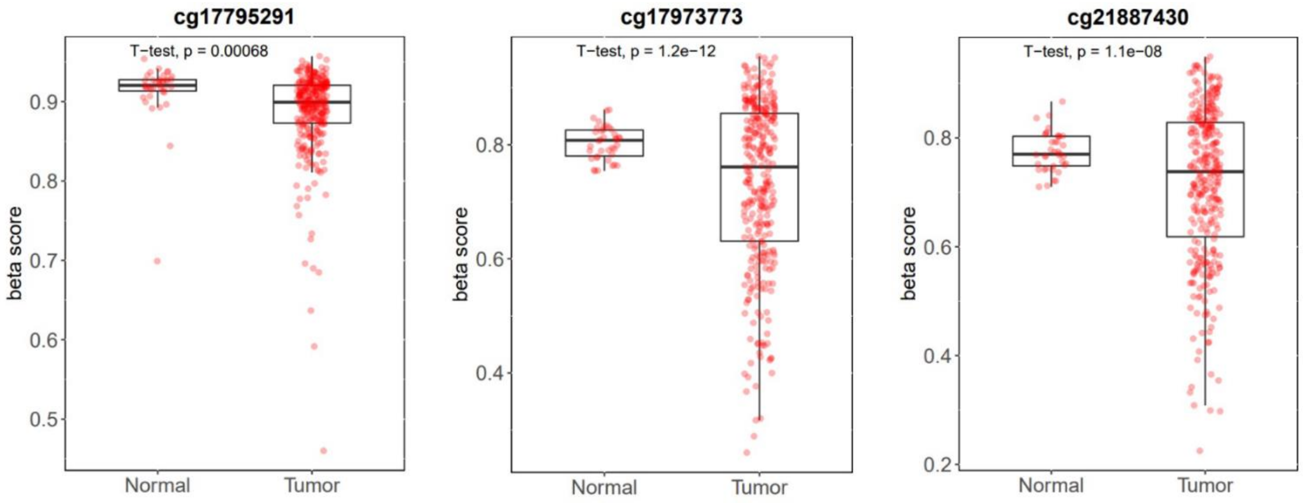

Supplementary Figure S3. The methylation status of *PBK* promoter region in TCGA COAD and matched normal samples. The beta-score for each sample was obtained from the Xena Browser. Statistical significance was determined by a two-tailed Student's *t*-test.

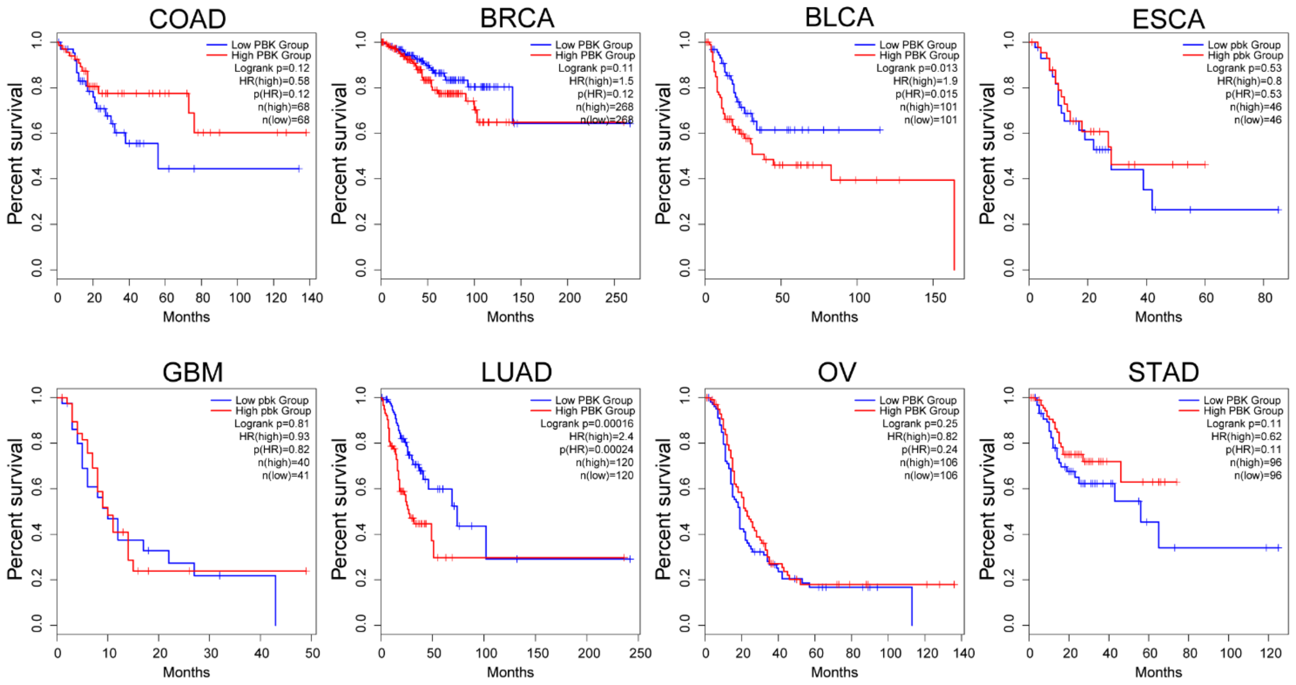

Supplementary Figure S4. Prognostic values of PBK/TOPK in various cancer types. Kaplan–Meier curves for disease-free survival (DFS) according to PBK/TOPK expression across different solid tumors in TCGA.

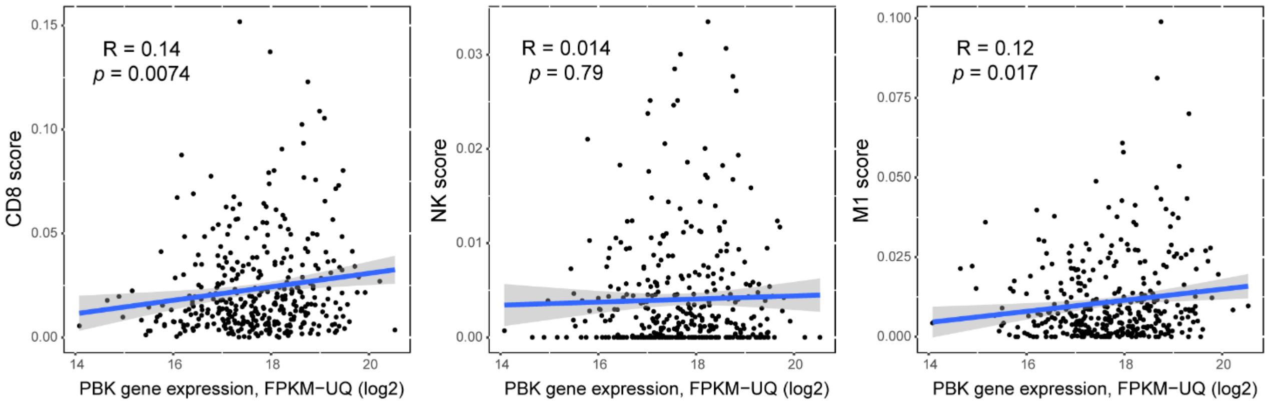

Supplementary Figure S5. Correlation between PBK/TOPK expression and immune cell infiltration in colon cancer. The CD8+ T cell, NK cell, and M1 macrophage scores were downloaded from Liu *et al.* [1]. Each score represents the CIBERSORT prediction score × leukocyte fraction. The PBK/TOPK gene expression data were downloaded from the Xena Browser. The correlations were visualized with dot plots using R software. Statistical significance was determined by Spearman's correlation *p* value.

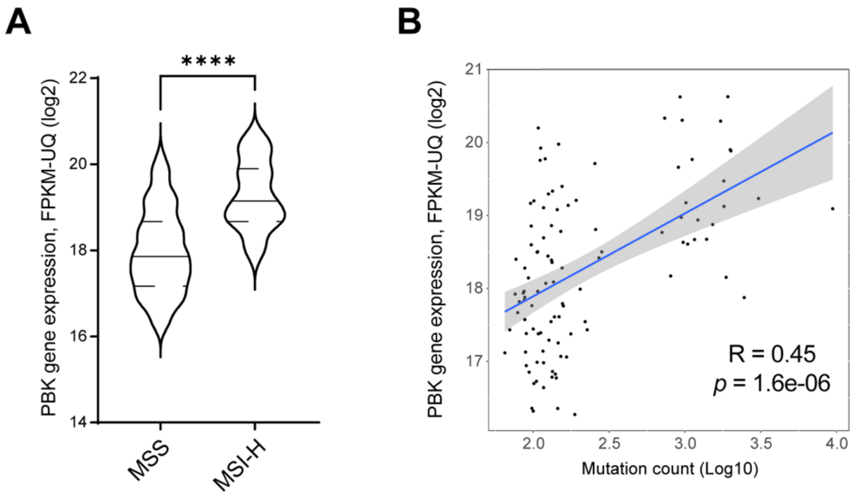

Supplementary Figure S6. Correlation between PBK/TOPK expression and MSI subtype in the CPTAC-2 colon cancer samples. PBK/TOPK gene expression, MSI status, and total mutation count data for the CPTAC-2 colon cancer samples were obtained from the GDC data portal. **(A)** Violin plots demonstrate PBK/TOPK gene expression by MSI status in the CPTAC-2 colon cancer samples. Statistical significance was determined by a two-tailed Student's *t*-test. **(B)** Dot plots represent the correlation between PBK/TOPK expression and total mutation count. Statistical significance was determined by Spearman's correlation *p* value.

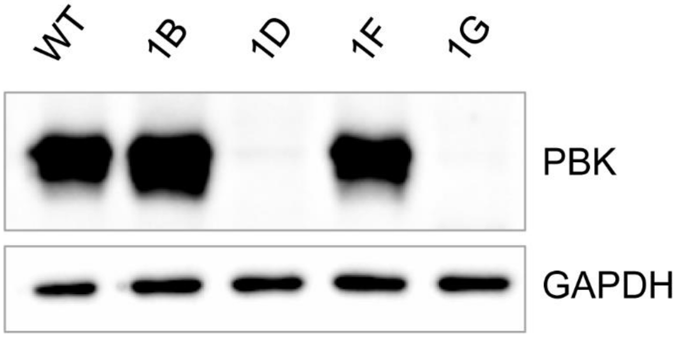

Supplementary Figure S7. PBK/TOPK gene knockout in the HCT-116 colon cancer cell line. Western blot image of PBK/TOPK in HCT-116 wild-type (WT) and single-cell knockout (KO) clones (1B, 1D, 1F, and 1G). GAPDH was used for the internal loading control. PBK/TOPK was completely deleted in clone numbers 1D and 1G. 1D clone was used for further analysis.

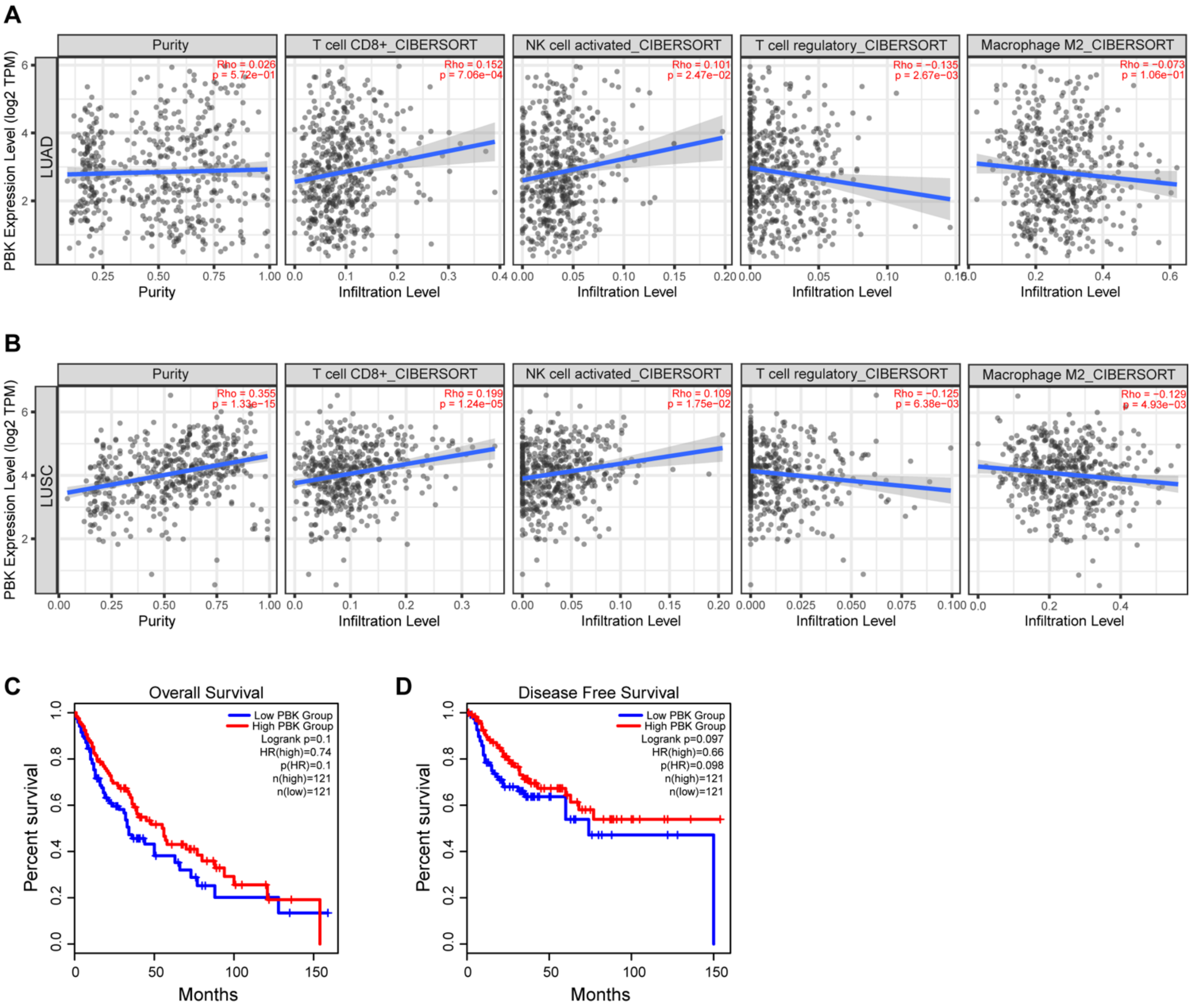

Supplementary Figure S8. Correlation of PBK/TOPK expression with immune cell infiltration level and prognosis in lung cancer. The correlation analysis between PBK/TOPK expression and immune cell infiltration was conducted with the TIMER2.0 web portal for the **(A)** lung adenocarcinoma (LUAD) and **(B)** lung squamous cell carcinoma (LUSC) samples in TCGA. Statistical significance was determined by Spearman's correlation *p* value. Kaplan–Meier curves for **(C)** OS and **(D)** DFS according to PBK/TOPK expression for LUSC samples in TCGA.

References

1. Liu, Y.; Sethi, N.S.; Hinoue, T.; Schneider, B.G.; Cherniack, A.D.; Sanchez-Vega, F.; Seoane, J.A.; Farshidfar, F.; Bowlby, R.; Islam, M.; et al. Comparative Molecular Analysis of Gastrointestinal Adenocarcinomas. *Cancer Cell* **2018**, *33*, 721-735 e728, doi:10.1016/j.ccell.2018.03.010.
